# The Clinical Profile and Bedside Clues of Anti‐LGI1 Encephalitis Presenting With Isolated Cognitive Impairment: A Multicenter Retrospective Cohort Study

**DOI:** 10.1002/cns.71258

**Published:** 2026-11-09

**Authors:** Yu Jia, Huifang Wang, Jinfeng Zhang, Qianqian Wang, Yingxue Yang, Lehong Gao, Wei Wang, Jia Chen, Yicong Lin, Haohao Kang, Xin Su, Zhaoyang Huang, Aihua Liu, Liankun Ren, Yuping Wang

**Affiliations:** ^1^ Department of Neurology Xuanwu Hospital, Capital Medical University Beijing China; ^2^ National Center for Neurological Disorders Beijing China; ^3^ Clinical Research Center of Epilepsy Xuanwu Hospital, Capital Medical University Beijing China; ^4^ Department of Neurology First Hospital of Shanxi Medical University Shanxi China; ^5^ Department of Neurology Baotou Central Hospital Baotou Inner Mongolia China

**Keywords:** anti‐LGI1 encephalitis, evolution, immunotherapy, isolated cognitive impairment, Montreal cognitive assessment (MoCA)

## Abstract

**Aims:**

A significant subset of anti‐LGI1 encephalitis manifests initially with isolated cognitive impairment. Characterizing the clinical profile and bedside clues is critical for timely diagnosis and treatment.

**Methods:**

This multicenter retrospective cohort study enrolled 43 patients with isolated cognitive impairment during the first 2 weeks of illness. Patients were classified according to their entire disease course into sustained isolated impairment versus evolving neuropsychiatric syndrome. Domain‐specific cognitive deficits (assessed using the Montreal Cognitive Assessment [MoCA]), paraclinical findings, and treatment response were systematically evaluated.

**Results:**

Median age was 58 years and 67.4% were male. The MoCA revealed delayed recall impairment in 93.0%, with relatively preserved naming. Additional neuropsychiatric symptoms developed in 69.8% of patients, with median latencies as follows: psychiatric symptoms (21 days), FBDS (30 days), and focal seizures (47.5 days). The remaining 30.2% suffered from a sustained isolated cognitive impairment. The sustained‐isolation subgroup was younger, had fewer naming deficits, and a similar immunotherapy response. EEG abnormalities (53.9%) and mesial temporal MRI hyperintensities (61.5%) were common in this subgroup.

**Conclusion:**

Isolated cognitive impairment frequently evolves into a broader neuropsychiatric syndrome, but a subset remains isolated. Delayed recall impairment with relative naming preservation, together with EEG and MRI findings, constitutes a practical bedside triad prompting early antibody screening and timely immunotherapy.

## Graphical abstract

Anti‐LGI1 encephalitis frequently presents with isolated cognitive impairment, with most patients evolving into a broader neuropsychiatric syndrome. A distinct subset maintains sustained isolated cognitive decline, characterized by delayed recall impairment, relative naming preservation, and frequent EEG and mesial temporal MRI abnormalities. Recognizing this bedside triad as suspicion‐raising clues may prompt early antibody screening and timely immunotherapy.

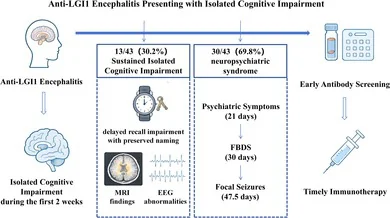

**Abbreviations**
Alb: albumin
APE2: antibody prevalence in epilepsy and encephalopathy
CASE: clinical assessment scale in autoimmune ncephalitis
CBA: cell‐based assays
CSF: cerebrospinal fluid
EEG: electroencephalogram
FBDS: faciobrachial dystonic seizures
FLAIR: fluid attenuated inversion recovery
IFA: immunofluorescence assay
IgG: immunoglobulin G
IQR: interquartile range
IVIg: intravenous immunoglobulin
LGI1: Leucine‐rich Glioma‐inactivated 1
MoCA: montreal cognitive assessment
MRI: magnetic resonance imaging
mRS: modified rankin scale
OCB: oligoclonal bands

## 1 Introduction

Cognitive impairment arises from diverse etiologies, including neurodegenerative diseases, metabolic disorders, stroke, and inflammatory conditions. Autoimmune encephalitides represent a particularly important subset of these disorders, as they constitute potentially reversible causes of cognitive decline [1, 2]. Anti‐leucine‐rich glioma‐inactivated 1 (LGI1) encephalitis, the second most common autoimmune encephalitis, is classically characterized by faciobrachial dystonic seizures (FBDS), psychiatric symptoms, rapidly progressive cognitive impairment, and refractory hyponatremia [3].

While the emergence of FBDS often facilitates the clinical recognition of LGI1 encephalitis, a substantial proportion of patients present with cognitive impairment as the initial manifestation. Cognitive deficits occur in 70%–90% of patients during the disease course but are sometimes the sole presenting feature in routine practice [3, 4]. Retrospective cohort studies suggest that approximately 10%–30% of patients may exhibit isolated cognitive decline without concomitant FBDS, epileptic seizures, or prominent psychiatric symptoms during the early disease phase; however, these estimates derive from single‐center series with heterogeneous operational definitions of “isolated,” and precise dating of symptom onset is frequently limited by the insidious nature of the disorder [5, 6, 7]. Crucially, even within this cognitive‐predominant subgroup, many patients evolve to develop typical neuropsychiatric manifestations over subsequent weeks, underscoring the challenge of distinguishing a genuinely isolated phenotype from a transient early phase of a broader syndrome. Whether patients in whom cognitive impairment is the initial manifestation demonstrate a distinct profile of domain‐specific cognitive deficits or a unique longitudinal trajectory therefore remains insufficiently characterized and represents a critical knowledge gap with direct implications for early diagnosis and treatment.

To address this issue, we conducted a multicenter retrospective cohort study of patients with LGI1 encephalitis who manifested as isolated cognitive impairment‐Onset phenotype. Rather than relying on short‐term symptomatic windows, we classified patients based on their entire clinical course: those with sustained isolated cognitive impairment versus those who subsequently evolved into a broader neuropsychiatric syndrome. The study systematically characterized domain‐specific cognitive deficits using the Montreal Cognitive Assessment (MoCA) scale [8]. We chose the MoCA as the primary instrument because, in real‐world acute neurology practice, patients presenting with isolated cognitive decline are routinely screened with MoCA rather than a 2–3 h neuropsychological battery. This maximizes generalizability and reflects the exact bedside scenario in which the diagnostic delay occurs. Then the temporal sequence and progression of clinical symptoms prior to diagnosis and treatment initiation were analyzed. This study aims to (1) delineate the natural history of the isolated cognitive‐onset phenotype, (2) identify bedside clues that facilitate early recognition, and (3) provide a framework for differentiating this treatable mimic of neurodegenerative dementia.

## 2 Methods

### 2.1 Study Design and Participants

This multicenter, retrospective cohort study consecutively enrolled patients diagnosed with anti‐LGI1 encephalitis at the Departments of Neurology of Xuanwu Hospital (Capital Medical University), Baotou Central Hospital, and the First Hospital of Shanxi Medical University between January 2023 and January 2024.

All participants fulfilled the diagnostic criteria for anti‐LGI1 encephalitis [9]. Inclusion criteria: (1) age ≥ 16 years; (2) cognitive impairment as the initial manifestation, with no other neurologic or psychiatric features (e.g., psychiatric symptoms, seizures, FBDS, language deficits, movement disorders) during the first 2 weeks after symptom onset. This 2 week isolated window served as an inclusion filter, not a classification criterion; (3) availability of complete MoCA scores, cerebrospinal fluid (CSF) analysis, and brain Magnetic Resonance Imaging (MRI) results obtained prior to the initiation of immunotherapy; (4) negative results of testing for 14–3‐3 protein in both serum and CSF, and no finding of pathogenic organisms on metagenomics next generation sequencing; and (5) no prior history of dementia.

During the study period, 125 patients with anti‐LGI1 encephalitis were screened for eligibility across the three participating centers (Xuanwu Hospital, *n* = 71; First Hospital of Shanxi Medical University, *n* = 33; Baotou Central Hospital, *n* = 21). Eighty‐two patients were excluded because they did not meet the inclusion criteria: 51 had cognitive impairment that was not isolated within the first 2 weeks of symptom onset, 21 lacked complete baseline data (MoCA, brain MRI, or CSF analysis) because these assessments were not performed during the index admission (*n* = 14), patients were transferred before completion (*n* = 2), or records were unavailable (*n* = 5); 4 had a prior history of dementia, and 6 had alternative diagnoses (including Creutzfeldt‐Jakob disease or viral encephalitis). The remaining 43 patients were finally enrolled (Xuanwu Hospital, *n* = 24; First Hospital of Shanxi Medical University, *n* = 12; Baotou Central Hospital, *n* = 7). The flow of patient selection is summarized in Figure S1. The 21 excluded patients due to missing data had a median age of 60 (interquartile range 15) years, 61.9% were male, and 71.4% had rapidly progressive cognitive decline. Compared with the 43 included patients, no significant differences were observed in age, sex, or proportion with rapidly progressive dementia (all *p* values > 0.05). Nevertheless, the potential for selection bias arising from complete‐case exclusion cannot be excluded and is acknowledged as a limitation.

Subgroup classification: After the initial 2 weeks window, patients were categorized into two groups based on their complete clinical course prior to last follow‐up: (1) sustained isolated cognitive impairment (no additional neuropsychiatric manifestations at any time), and (2) evolving neuropsychiatric phenotype (development of psychiatric symptoms, seizures, FBDS, or other neurological deficits after the first 2 weeks).

### 2.2 Clinical and Neuropsychological Assessment

Cognitive impairment was clinically diagnosed according to standard criteria, requiring evidence of decline in memory and at least one other cognitive domain, along with interference in daily functioning persisting for more than 2 weeks [10, 11]. In this acute neuroinflammatory context, the term “dementia” was applied clinically rather than implying an irreversible neurodegenerative process. Global and domain‐specific cognitive function were assessed using MoCA, which evaluates visuospatial ability, executive function, naming, attention, language, abstraction, memory, and orientation [8]. The MoCA is a validated screening instrument for cognitive impairment in autoimmune encephalitis and is widely used clinically due to its brevity and multidimensional assessment capability, making it a practical tool for initial evaluation. The total possible score is 30, and one point was added for participants with ≤ 12 years of formal education to correct for educational bias. Given the acuity of presentation, MoCA cutoffs were used to stratify severity: Mild (18–25), moderate (10–17), and severe (< 10) [8]. Patients with MoCA scores in the mild range (18–25) met criteria for clinically significant cognitive impairment impacting daily activities, consistent with the concept of “reversible dementia” in autoimmune encephalitis [5, 7], a phenotype increasingly recognized as a treatable mimic of neurodegenerative disorders. Of note, MoCA was the only standardized cognitive instrument administered at baseline in all 43 patients; detailed domain‐specific neuropsychological testing (e.g., list‐learning tasks, semantic fluency norms, executive batteries) was not uniformly available in the three retrospective centers and thus not used as a primary endpoint. This constrained the depth of cognitive profiling but ensured completeness of data across the whole cohort.

All patients underwent continuous Electroencephalogram (EEG) monitoring for a minimum of 2 h during their hospitalization; 12 patients (27.9%) had prolonged monitoring lasting ≥ 4 h. All recordings were reviewed by experienced clinical neurophysiologists at each center. EEG abnormalities were categorized as (1) slowing alone, (2) isolated epileptiform discharges, or (3) slowing with epileptiform discharges.

### 2.3 Antibody Testing and CSF Analysis

The Antibody Prevalence in Epilepsy and Encephalopathy (APE2) scores were calculated prior to antibody testing to estimate the probability of detecting autoimmune antibodies [12].

All patients were tested for IgG autoantibodies associated with autoimmune encephalitis. Screening was performed using indirect immunofluorescence assay (IFA; EUROIMMUN, FA112d‐1), with positive results confirmed by commercial cell‐based assays (CBA; Euroimmun) prior to immunotherapy initiation. The antibody panel included autoimmune encephalitis and onconeural antibodies. Inclusion required positivity for LGI1 antibodies in both serum and CSF, with negativity for all other tested antibodies (Figure S2). The initial dilution titers for serum and CSF were 1:10 and 1:1, respectively. CSF antibody titers were categorized as weakly positive (1:1), moderately positive (1:3.2 to 1:10), or strongly positive (≥ 1:32) [13].

Standard CSF analysis included cell count, protein concentration, IgG index, and oligoclonal bands (OCB). Pleocytosis was defined as a white cell count > 5 cells/μL, and elevated protein as a concentration > 50 mg/dL. An IgG index (Q_Immunoglobulin G_/Q_Albumin_ [Q_IgG_/Q_Alb_]) > 0.7 was considered elevated [14].

### 2.4 Outcome Measures and Definitions

Clinical outcomes were assessed at admission and at two follow‐up timepoints (6 months and 1 year after discharge) using the modified Rankin Scale (mRS) and the Clinical Assessment Scale in Autoimmune Encephalitis (CASE). The CASE score evaluates nine symptom domains: seizures, memory, psychiatric features, consciousness, language, movement disorders, gait/ataxia, brainstem function, and weakness. Each domain is scored from 0 to 3, yielding a total score ranging from 0 to 27 [15]. The mRS score ranges from 0 (no symptoms) to 6 (death). Treatment responders were defined as patients who achieved either a ≥ 50% reduction in the total CASE score or a ≥ 1 point in the mRS score from baseline, consistent with prior autoimmune encephalitis studies [15, 16]. Disease relapse was defined as the emergence or worsening of neurological symptoms after at least 2 months of sustained clinical improvement or stability [9]. Functional outcome was assessed using the mRS at 12 months. While previous consensus recommendations and autoimmune encephalitis outcome studies commonly define a “good outcome” as mRS ≤ 2 [15, 16], we employed a stricter definition of mRS ≤ 1 as the threshold for favorable recovery. This decision reflects the recognition that LGI1 encephalitis predominantly manifests with cognitive, psychiatric, and seizure‐related morbidity rather than gross motor deficits; consequently, patients with mRS = 2 (minor disability) may still harbor clinically significant memory impairment or personality changes that preclude return to baseline functioning. Therefore, in this cohort, a “good outcome” was defined as mRS ≤ 1, whereas a “poor outcome” was defined as mRS ≥ 2.

### 2.5 Statistical Analysis

Statistical analyses were performed using IBM SPSS Statistics software (version 23.0). Continuous variables are presented as median with interquartile range (interquartile range, IQR), and categorical variables as frequency (percentage, %). Between‐group comparisons were conducted using Pearson's *χ* ^2^ test, Fisher's exact test, or the Mann–Whitney *U* test, as appropriate. A two‐tailed *p* value < 0.05 was considered statistically significant.

## 3 Results

### 3.1 Patient Characteristics and Domain‐Specific Cognitive Impairment Profile

Forty‐three patients were enrolled, including 29 males (67.4%), with a median age at symptom onset of 58 years (IQR 17, range 23–81) (Table S1). The median diagnostic delay was 50 days (IQR 70, range 14–270). Rapidly progressive dementia was observed in 31 patients (72.1%). The median MoCA score at diagnosis was 17 (IQR 9). Cognitive impairment was mild in 21 patients (48.8%), moderate in 17 (39.5%), and severe in 5 (11.6%). The MoCA revealed that deficits in delayed recall (93.0%) and orientation to time/place (60.5%) were the most frequent findings, whereas impairments in abstraction and naming were less common (Table 1). Notably, 26 patients (60.5%) exhibited deficits in three or more cognitive domains (Table S2).

**TABLE 1:** Domain‐specific cognitive deficits in LGI1 encephalitis assessed by the montreal cognitive assessment (MoCA) scale.

Impaired cognitive domains	Number (%)
Visuospatial abilities	18 (41.9%)
Executive function	16 (37.2%)
Naming	11 (25.6%)
Attention	24 (55.8%)
Language	10 (23.3%)
Abstraction	8 (18.6%)
Delayed Recall	40 (93.0%)
Orientation	26 (60.5%)

### 3.2 Temporal Evolution of Neuropsychiatric Symptoms

Thirteen patients (30.2%) exhibited isolated cognitive impairment throughout their disease course, with a median time to diagnosis of 30 days (IQR 32, range 15–120) from symptom onset. The remaining 30 patients (69.8%) developed additional neuropsychiatric symptoms, including psychiatric features, focal seizures, faciobrachial dystonic seizures (FBDS), language disturbances, and movement disorders.

Psychiatric symptoms emerged in 17 patients (39.5%) at a median latency of 21 days (IQR 16, range 14–60) after onset, manifesting as abnormal behavior (*n* = 14, 82.4%), psychosis (*n* = 10, 58.8%), mood disorders (*n* = 8, 47.1%), and catatonia (*n* = 3, 17.7%). FBDS developed in 11 patients (25.6%) at a median of 30 days (IQR 32, range 14–76). Focal seizures occurred in 14 patients (32.6%) at a median latency of 47.5 days (IQR 46.5, range 14–120), with presentations including behavioral arrest (*n* = 7, 50.0%), clonic seizures (*n* = 7, 50.0%), focal to bilateral tonic–clonic seizures (*n* = 6, 42.9%), and less commonly emotional, sensory, or autonomic seizures. Language deficits appeared in 5 patients (11.6%) at a median of 28 days (IQR 11.5, range 21–42), comprising verbal fluency deficits (*n* = 3, 60.0%) and aphasia (*n* = 2, 40.0%). Movement disorders (characterized by involuntary movements of the lips and limbs) were observed in 3 patients (7.0%) at a median of 42 days (range 30–45).

Less prominent features included autonomic dysfunction in 7 patients (16.3%), primarily hyperhidrosis, and sleep disorders in 16 (37.2%), manifesting as hypersomnia, insomnia, or dream enactment behaviors. A preceding infection within 2 weeks prior to symptom onset was reported in 5 patients (11.6%), with fever, headache, and cough being the most common features.

### 3.3 Laboratory, Imaging, and Electrophysiologic Findings

The mean APE2 score was 5.0 ± 2.1 (range 1–10), with 31 patients (72.1%) scoring ≥ 4. Hyponatremia was observed in 9 patients (20.9%). CSF analysis revealed pleocytosis in 7 patients (16.3%), elevated protein in 7 (16.3%), an elevated IgG index in 6 (14.0%), and positive OCB in 2 (4.7%). LGI1 antibody titers were weakly positive in 11 patients (25.6%), moderately positive in 11 (25.6%), and strongly positive in 21 (48.8%) (Table S2). The results of brain MRI showed different abnormal patterns (Figure 1). Mesiotemporal T2/Fluid Attenuated Inversion Recovery (FLAIR) hyperintensities were found in 21 patients (48.8%), which were unilateral in 7 (left: 5, right: 2) and bilateral in 14. Extramesiotemporal involvement was noted in 2 patients (4.7%). Hippocampal atrophy was present in 6 patients (14.0%). EEG abnormalities were observed in a significant proportion of patients and included slowing with epileptiform discharges in 8 patients (18.6%), isolated epileptiform discharges in 9 (20.9%), and slowing alone in 8 (18.6%). Two patients (4.7%) had positive paraneoplastic antibody screening.

**FIGURE 1:**
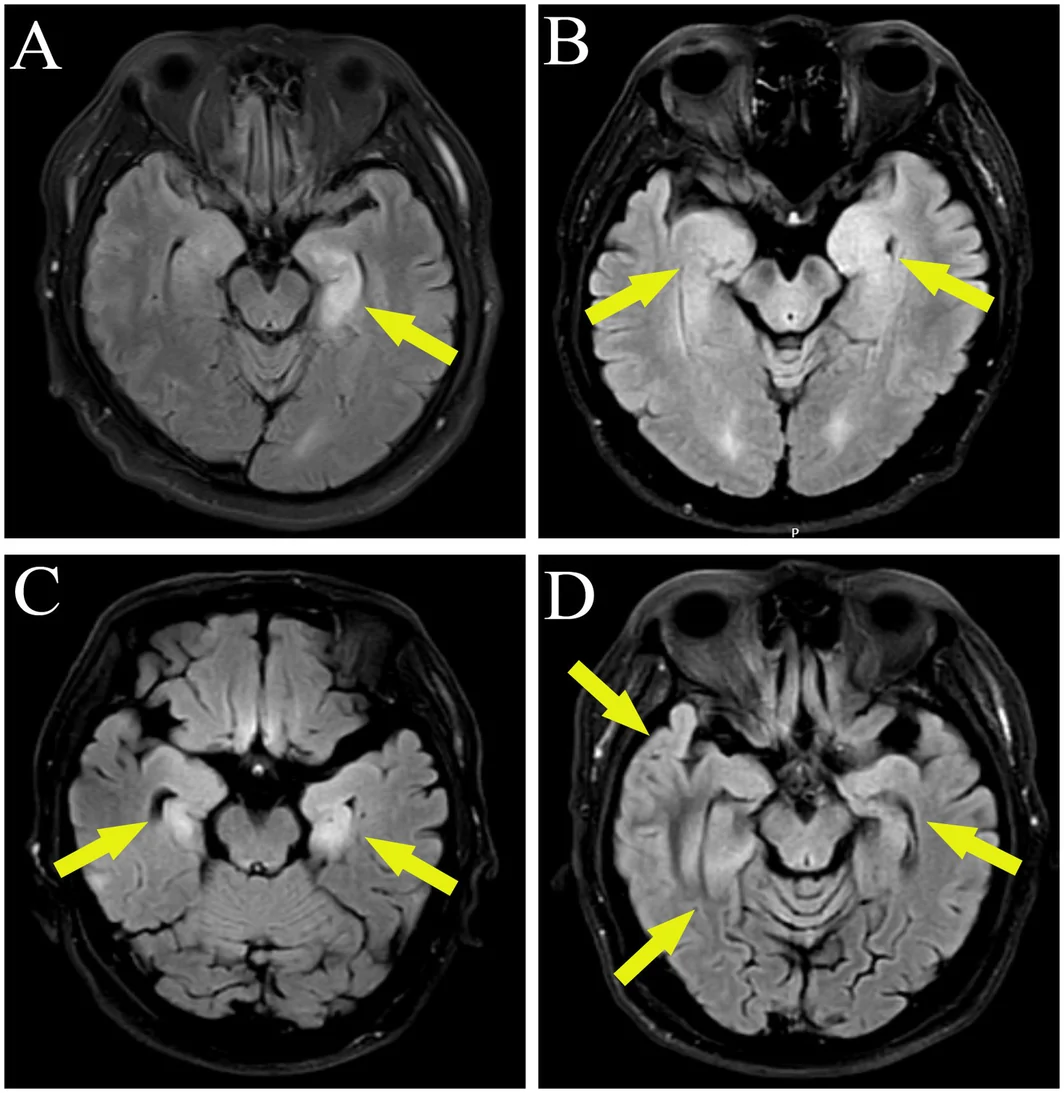
Brain MRI findings in anti‐LGI1 encephalitis with cognitive impairment as the initial manifestation. Representative axial FLAIR sequences on brain MRI showing four distinct patterns of imaging abnormalities in patients in the cohort study. (A) Unilateral hyperintensity in the left mesial temporal lobe (arrow). (B) Bilateral hyperintensity with swelling in the mesial temporal lobes (arrows). (C) Bilateral mesial temporal lobe hyperintensity accompanied by right hippocampal atrophy (arrow). (D) Involvement of bilateral mesial temporal lobes, extending to the right lateral temporal lobe and right frontal lobe, with diffuse cortical atrophy (arrows). All images are presented on a black background to optimize contrast for structural visualization.

### 3.4 Treatment Response and Clinical Outcomes

All patients received first‐line immunotherapy, consisting of glucocorticoids, intravenous immunoglobulin (IVIg), or a combination of both. Among these, 34 patients (79.1%) responded to first‐line therapy, with 30 (88.2% of responders) improving within 4 weeks. Thirteen patients (30.2%) required second‐line immunotherapy due to an insufficient response to first‐line treatment, of whom 6 (46.2%) subsequently improved. At the 12 months follow‐up visit, 36 patients (83.7%) attained a good outcome, and 7 (16.3%) had poor outcomes (Figure 2). Relapse occurred in 3 patients (7.0%), all of whom exhibited milder severity compared to the initial episode.

**FIGURE 2:**
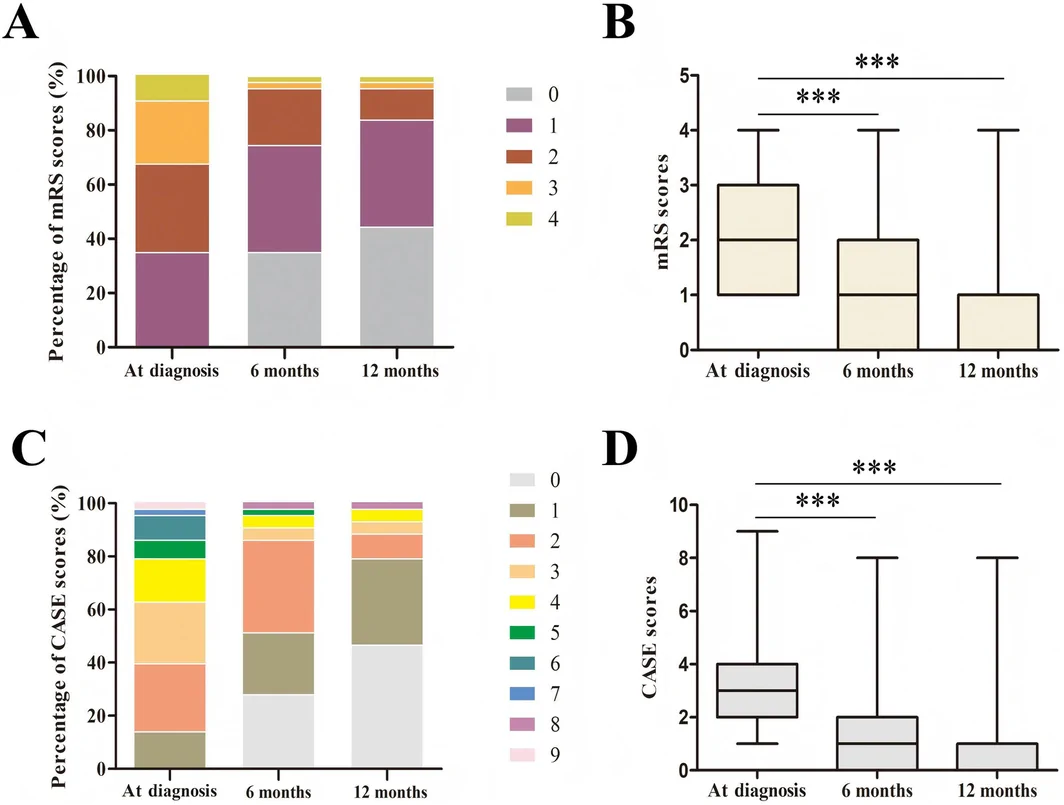
Longitudinal comparison of functional outcomes (mRS) and clinical severity (CASE) scores at time of diagnosis, 6 months, and 12 months after treatment. This composite figure illustrates the distribution of the modified Rankin Scale (mRS) and CASE scores at three time points: Time of diagnosis, 6 months, and 12 months after treatment. (A) Stacked bar chart showing the percentage distribution of mRS scores (0–6). Over time, the proportion of patients with favorable outcomes (mRS 0–2) increases, while the proportion with higher scores (worse disability) decreases. (B) Box‐and‐whisker plot of the mRS scores, confirming a significant reduction in the median score over time. Statistical significance (*p* < 0.001) between time points is indicated by “***”. (C) Stacked bar chart showing the percentage distribution of CASE scores (0–9). The distribution shifts toward lower (less severe) scores at 6 and 12 months compared to the time of diagnosis. (D) Box‐and‐whisker plot of the CASE scores, showing a significant decline in median severity scores over the follow‐up period. Overall, the figure demonstrates significant clinical improvement in both functional status (mRS) and disease‐specific severity (CASE) from the time of diagnosis to 6‐ and 12 months follow‐ups.

### 3.5 Subgroup Analysis: Clinical Features and Outcomes in Patients

As shown in Table 2, no significant differences in clinical characteristics were found between poor‐outcome (*n* = 7) and good‐outcome (*n* = 36) groups (all *p* values > 0.05). A subset of patients with the seemingly less severe “pure cognitive” phenotype still progressed to a poor outcome. Based on the entire disease course, 13 patients (30.2%) exhibited sustained isolated cognitive impairment, whereas 30 patients (69.8%) evolved into a broader neuropsychiatric syndrome (Table 3). Compared with the evolving‐phenotype group, patients with sustained isolated cognitive impairment were younger (median 52 vs. 62 years, *p* = 0.03) and had a lower frequency of naming deficits (15.4% vs. 46.7%, *p* = 0.04).

**TABLE 2:** Comparative analysis of patients with Anti‐LGI1 encephalitis: Good recovery versus Poor recovery at the 12 months follow‐up visit.

Variable		LGI encephalitis presenting cognitive impairment		*p*
		Good recovery (*n* = 36)	Poor recovery (*n* = 7)	
Age at onset (years), [M (IQR)]		57.5 (17.5)	67 (16)	0.343
Delay treatment (days), [M (IQR)]		45 (79)	60 (30)	0.451
Gender (male), *N* (%)		25 (69.4%)	4 (57.1%)	0.846
Isolated cognitive impairment, *N* (%)		12 (33.3%)	1 (14.3%)	0.579
Impaired Cognitive Domains, *N* (%)				
Visuospatial abilities		13 (36.1%)	5 (71.4%)	0.189
Executive function		12 (33.3%)	4 (57.1%)	0.444
Naming		8 (19.4%)	3 (42.9%)	0.502
Attention		18 (50.0%)	6 (85.7%)	0.185
Language		7 (19.4%)	2 (28.6%)	0.972
Abstraction		6 (16.7%)	2 (28.6%)	0.834
Delayed Recall		33 (91.7%)	7 (100%)	1.000
Orientation		22 (61.1%)	4 (57.1%)	1.000
Three or more domains deficits		21 (58.3%)	5 (71.4%)	0.821
Scale scores at diagnosis				
MOCA, [M (IQR)]		17 (8.75)	18 (12)	0.133
Degree of cognitive impairment (MOCA), N (%)	Mild cognitive impairment (18–25 points)	17 (47.2%)	2 (28.6%)	0.622
	Moderate cognitive impairment (10–17 points)	14 (38.9%)	4 (57.1%)	0.633
	Severe cognitive impairment (Under 10 points)	5 (13.9%)	1 (14.3%)	1.000
CASE, [M (IQR)]		3 (2)	4 (3)	0.277
mRS, [M (IQR)]		2 (2)	2 (2)	0.800
Auxiliary examination, N (%)				
CSF inflammatory abnormality^a^		12 (33.3%)	2 (28.6%)	1.000
Antibody levels in CSF, N (%)	Weakly positive	8 (15.4%)	3 (42.9%)	0.502
	Moderately positive	8 (22.2%)	3 (42.9%)	0.502
	Strongly positive	20 (55.6%)	1 (14.3%)	0.113
Brain MRI abnormality, N (%)	Suggesting encephalitis^b^	19 (52.8%)	2 (28.6%)	0.448
	Hippocampal atrophy	4 (11.1%)	2 (28.6%)	0.248
EEG abnormality, N (%)	Slow wave	13 (36.1%)	3 (42.9%)	1.000
	Epileptic discharge	13 (36.1%)	4 (57.1%)	0.536

^a^ CSF inflammatory abnormality was defined as is defined as the presence of at least one of the following abnormalities, including pleocytosis, elevated protein concentration, elevated immunoglobulin G index, or positive oligoclonal bands.

^b^ Brain MRI hyperintense signals on T2WI/FLAIR sequences restricted to one or both medial temporal lobes or in multifocal areas involving gray matter, which highly suggested the features of autoimmune encephalitis.

**TABLE 3:** Comparative analysis of patients with anti‐LGI1 encephalitis: Isolated cognitive impairment versus cognitive impairment with neuropsychiatric features.

Variable		LGI encephalitis presenting cognitive impairment		*p*
		Isolated cognitive impairment (*n* = 13)	Accompanying with neuropsychiatric symptoms (*n* = 30)	
Age at onset (years) [M (IQR)]		52 (23)	62 (13.75)	**0.005**
Gender (male), *N* (%)		9 (69.2%)	20 (66.7%)	1.000
Impaired Cognitive Domains, *N* (%)				
Visuospatial abilities		6 (46.2%)	12 (40%)	0.747
Executive function		6 (46.2%)	10 (33.3%)	0.649
Naming		0	11 (36.7%)	**0.032**
Attention		6 (46.2%)	18 (60.0%)	0.509
Language		5 (38.5%)	5 (16.7%)	0.246
Abstraction		1 (7.7%)	7 (23.3%)	0.433
Delayed recall		12 (92.3%)	28 (93.3%)	1.000
Orientation		8 (61.5%)	18 (60%)	1.000
Three or more domains deficits		9 (69.2%)	17 (56.7%)	0.513
Scale scores at diagnosis				
MOCA, [M (IQR)]		21 (8)	17 (10.25)	0.133
Degree of cognitive impairment (MOCA), *N* (%)	Mild cognitive impairment (18–25 points)	6 (46.2%)	13 (43.3%)	1.000
	Moderate cognitive impairment (10–17 points)	6 (46.2%)	12 (40.0%)	0.747
	Severe cognitive impairment (Under 10 points)	1 (7.7%)	5 (16.7%)	0.764
APE2	APE2 scores	3.0 ± 1.2	5.8 ± 1.9	**< 0.01**
	APE2 ≥ 4, *N* (%)	2 (15.4%)	29 (96.7%)	**< 0.01**
Auxiliary examination, *N* (%)				
CSF inflammatory abnormality^a^		4 (30.8%)	10 (33.3%)	1.000
Antibody levels in CSF, *N* (%)				
Antibody levels in CSF, *N* (%)	Weakly positive	2 (15.4%)	9 (30.0%)	0.530
	Moderately positive	3 (23.1%)	8 (26.7%)	1.000
	Strongly positive	8 (61.5%)	13 (43.3%)	0.332
Brain MRI abnormality, *N* (%)	Suggesting encephalitis^b^	8 (61.5%)	13 (43.3%)	0.332
	Hippocampal atrophy	2 (15.4%)	4 (13.3%)	1.000
EEG abnormality, *N* (%)	Slow wave	3 (23.1%)	13 (43.3%)	0.358
	Epileptic discharge	4 (30.8%)	13 (43.3%)	0.513
Outcomes at the 12‐months follow‐up visit, *N* (%)				
Good recovery		11 (84.6%)	25 (83.3%)	1.000
Poor recovery		2 (15.4%)	5 (16.6%)	1.000

*Note:* Bold values indicate a statistically significant difference (*p* < 0.05).

^a^ CSF inflammatory abnormality was defined as the presence of at least one of the following abnormalities, including pleocytosis, elevated protein concentration, elevated immunoglobulin G index, or positive oligoclonal bands.

^b^ Brain MRI hyperintense signals on T2WI/FLAIR sequences restricted to one or both medial temporal lobes or in multifocal areas involving gray matter, which highly suggested the features of autoimmune encephalitis.

Notably, despite the absence of seizures or prominent psychiatric symptoms at any point, paraclinical abnormalities were common in the sustained‐isolation subgroup. Brain MRI revealed mesial temporal hyperintensities suggesting encephalitis in 8/13 patients (61.5%). EEG abnormalities were observed in 7/13 patients (53.9%), comprising slow waves in 3 (23.1%) and epileptic discharges in 4 (30.8%). These findings underscore the high diagnostic yield of EEG and brain MRI in patients presenting with apparently isolated cognitive decline and support their role as practical bedside clues prompting early antibody screening. No significant differences were observed in baseline MoCA scores, overall MRI or EEG abnormality rates compared with the evolving‐phenotype group, or in 12 months functional outcomes.

Given the exploratory nature of these analyses and the absence of correction for multiple comparisons, all subgroup findings should be interpreted as hypothesis‐generating rather than confirmatory. Moreover, because this cohort included only LGI1‐antibody‐positive patients without an antibody‐negative comparison group, the diagnostic performance of these features could not be quantified; they should therefore be viewed as suspicion‐raising clues warranting confirmatory testing rather than as definitive diagnostic criteria.

## 4 Discussion

This study provided a comprehensive clinical analysis of 43 patients with anti‐LGI1 encephalitis, enhancing and expanding the understanding of this autoimmune disorder. The findings highlighted the predominance of cognitive impairment as a core initial symptom, detailed the subsequent spectrum of neuropsychiatric manifestations, and identified a clinically significant subgroup characterized by isolated cognitive decline—a presentation prone to diagnostic delay. Additionally, we reported treatment responses, recurrence rates, and long‐term outcomes, while exploring factors associated with unfavorable prognosis. Overall, the data demonstrated that although the disease often responds to immunotherapy, significant diagnostic challenges exist in the early stages when cognitive symptoms predominate, necessitating high clinical vigilance to avoid delays in diagnosis and management.

While prior studies have acknowledged that anti‐LGI1 encephalitis may begin with cognitive complaints, most investigations have focused on patients presenting with FBDS or psychiatric features, and few have systematically characterized the isolated cognitive‐onset phenotype as a distinct clinical entity [5, 7]. Crucially, previous comparisons often relied on arbitrary short‐term symptomatic windows (e.g., 2 week cutoffs), which are vulnerable to recall bias and imprecise onset dating in this insidiously progressive disease. By classifying patients according to their full clinical trajectory, we provide a more ecologically valid delineation of two phenotypes: A sustained isolated cognitive impairment subgroup and an evolving neuropsychiatric subgroup. We show that the former is not merely a transient early phase but represents a stable, clinically deceptive phenotype with distinct demographic and cognitive features. Furthermore, by leveraging the MoCA—a ubiquitous bedside tool—we translate detailed‐battery insights from prior imaging studies into a practical diagnostic triad (delayed recall impairment, naming preservation, and paraclinical abnormalities), thereby bridging the gap between academic neuropsychology and everyday clinical decision‐making.

A key finding in this cohort was that all patients presented with isolated cognitive decline as the initial symptom. The cognitive profile, identified through MoCA subscale analysis, offered valuable diagnostic clues. The MoCA effectively mapped the cognitive profile. The near‐universal impairment in delayed recall and the high prevalence of disorientation closely align with the known predilection of LGI1 encephalitis for limbic structures, especially the hippocampus [4, 6]. MRI studies corroborated bilateral hippocampal atrophy with predominant involvement of CA2/3 and CA4/dentate gyrus subfields, which correlated strongly with verbal and visuospatial memory deficits [17, 18]. This pattern resulted in a profound anterograde amnesia, often accompanied by retrograde amnesia and disorientation, presenting as a classic hippocampal amnesia syndrome [19]. Notably, focal CA3 atrophy had been specifically linked to severe episodic autobiographical memory loss, while semantic memory remained relatively preserved, underscoring the selectivity of hippocampal damage [18]. In contrast, the relatively preserved naming function and abstract reasoning might indicate less involvement of the lateral temporal neocortex and frontal executive networks in the early stages [20]. This neuropsychological profile, characterized by severe episodic memory impairment with relative preservation of language and reasoning in the acute stage, might provide clinical clues to differentiate anti‐LGI1 encephalitis from early‐stage Alzheimer's disease and frontotemporal dementia [21, 22]. The presence of impairment in three or more cognitive domains in 60.5% of patients highlighted the diffuse yet uneven impact of the inflammatory process, supporting the concept of antibody‐mediated widespread synaptic dysfunction [23, 24]. A recognized limitation of this study is that cognitive phenotyping relied solely on the MoCA, a bedside screening instrument rather than a comprehensive neuropsychological battery [8]. Consequently, MoCA cannot resolve construct‐specific memory subsystems (free recall vs. recognition, source memory, working memory, autobiographical temporality) or disentangle episodic from semantic memory. The near‐universal delayed‐recall impairment (93.0%) and infrequent naming deficits observed herein are therefore consistent with, but do not prove, a CA3/dentate‐predominant hippocampal process reported in prior detailed‐battery and 7.0‐T MRI studies [17, 18]. These findings should not be interpreted as direct evidence of subfield‐level damage. Complementarily, Ariño et al. [25] reported that only 35% of 76 patients returned to baseline cognition at 2 years follow‐up, indicating network‐level memory disability may persist despite normalization of brief screening instruments. These considerations underscore the need for prospective studies pairing high‐resolution hippocampal MRI with a modular neuropsychological battery to refine the cognitive fingerprint of this syndrome.

Another contribution of this study was the characterization of the 13 patients with “isolated cognitive impairment” throughout their disease course. This phenotype was particularly deceptive and carries a high risk of misdiagnosis as a primary neurodegenerative or psychiatric disorder [7]. Notably, these patients exhibited neither the typical FBDS nor the prominent psychiatric symptoms that often facilitate diagnosis. Consequently, their time to diagnosis was shorter, a seemingly paradoxical finding that may reflect a more direct pathway toward autoimmune diagnostic testing after exclusion of common neurodegenerative and infectious etiologies, compared to patients who are incorrectly diverted to psychiatric services [7]. This subgroup exhibited a lower frequency of naming deficits, which might indicate a more restricted limbic encephalitis without involvement of the lateral temporal regions responsible for semantic processing. Importantly, despite the absence of seizures or prominent psychiatric symptoms at any point, paraclinical abnormalities were common in this sustained‐isolation subgroup. Brain MRI revealed mesial temporal hyperintensities suggesting encephalitis in 61.5% of patients. EEG abnormalities were observed in 53.9% of patients. These findings underscore the high diagnostic yield of EEG and brain MRI in patients presenting with apparently isolated cognitive decline and support their role as practical bedside clues prompting early antibody screening. The presence of epileptiform discharges on EEG was an interesting and important finding. While expected in patients with overt epileptic seizures, their presence in this clinically restricted subgroup suggests underlying, subclinical epileptogenic activity [26, 27, 28]. Collectively, relative preservation of naming, and a high prevalence of EEG abnormalities in this subgroup may serve as clinical alerts prompting consideration of anti‐LGI1 encephalitis in patients presenting with isolated cognitive decline. However, it is crucial to emphasize that these features were identified in a cohort restricted to LGI1‐antibody‐positive patients without an antibody‐negative comparison group. Therefore, the diagnostic performance (e.g., sensitivity, specificity) of this proposed bedside triad could not be quantified. The features should be interpreted as suspicion‐raising clues that warrant confirmatory antibody testing, rather than as definitive diagnostic criteria.

In contrast to the sustained‐isolation subgroup, the majority of patients (69.8%) exhibited a predictable, stepwise expansion of neuropsychiatric manifestations. The predictable evolution, beginning with cognitive decline and progressing to psychiatric symptoms and then to seizures (including FBDS), reflected a characteristic, though nonspecific, progression of limbic and paralimbic cortical dysfunction [29]. A small proportion exhibited hippocampal atrophy on MRI at the time of diagnosis, which might reflect a late sequela of chronic inflammation and be associated with later disease stages or delayed treatment [30]. However, a normal brain MRI did not exclude the diagnosis [31], ultra‐high‐resolution 7.0 T MRI could detect focal CA3 atrophy even when conventional MRI appears normal, highlighting the potential of advanced imaging techniques in detecting hidden lesions of anti‐LGI1 encephalitis [18]. Moreover, previous studies had indicated that specific brain imaging features, such as hyperintensities confined to the temporal lobes and less frequently exhibited swelling, might help distinguish LGI1 encephalitis from viral encephalitis and CJD [31]. However, the present study identified involvement beyond the temporal lobes in a minority of patients, highlighting the need for continued clinical vigilance.

Turning to treatment outcomes, the high response rate to immunotherapy supported the efficacy of a stepwise immunotherapeutic approach [16, 32]. Poor prognosis occurred in 16.3% of patients, warranting further investigation. Patients with poor response showed no differences in initial clinical severity or overall disease burden, assessed by MoCA, CASE, and mRS scores. Previous studies found delayed immunotherapy, resulting in a prolonged untreated inflammatory phase, might lead to irreversible neuronal loss, particularly hippocampal sclerosis, and was correlated with the severity of memory deficits [17, 33]. Another study revealed that bilateral hippocampal hyperintensity on initial MRI and lower premorbid cognitive reserve are independent predictors of long‐term cognitive impairment [6]. Additionally, the poor‐prognosis group might harbor more severe or extensive damage within critical hippocampal‐cortical networks [17, 18]. Hippocampal microstructural integrity (assessed by mean diffusivity on DTI) was impaired and correlated with cognitive performance, providing a potential imaging biomarker for network damage [17]. Future studies should determine the key targets of LGI1 antibody‐induced damage to neural networks through detailed longitudinal neuropsychological assessments and neuroimaging techniques, providing a foundation for the development of precise treatment [34]. It is important to acknowledge that the MoCA was not systematically repeated as a longitudinal outcome measure in this cohort. While useful for initial cognitive profiling, LGI1 encephalitis manifests with a heterogeneous neuropsychiatric spectrum that extends beyond isolated cognitive impairment. Consequently, global functional scales, specifically the mRS and CASE, were prioritized as primary endpoints at 6 and 12 months. Nevertheless, available follow‐up MoCA data suggested persistent memory impairment in a subset of individuals despite functional independence (mRS ≤ 1), underscoring that normalization of global disability scales does not equate to full cognitive restoration.

## 5 Limitations

First, although this cohort (*n* = 43) is among the largest focusing on the isolated cognitive‐onset phenotype, the sustained‐isolation subgroup (*n* = 13) remains modest. Consequently, all subgroup analyses—including comparisons of demographic, cognitive, and paraclinical features—should be interpreted as hypothesis‐generating rather than confirmatory. Additionally, because this study included only LGI1‐antibody‐positive patients without an antibody‐negative comparison group, the diagnostic performance of the proposed bedside triad (delayed recall impairment, relative naming preservation, and paraclinical abnormalities) could not be quantified; these features should therefore be viewed as suspicion‐raising clues rather than validated diagnostic criteria. Second, cognitive phenotyping relied exclusively on the MoCA, a bedside screening instrument rather than a comprehensive neuropsychological battery. While this reflects real‐world acute‐care practice and ensured complete baseline data across all patients, MoCA cannot resolve subdomain‐specific memory constructs. Detailed characterization of memory subtypes and their relationship to hippocampal subfield damage requires prospective studies incorporating modular neuropsychological testing alongside high‐resolution MRI. Third, twenty‐one patients were excluded due to incomplete baseline data. Although comparison of available baseline characteristics revealed no significant differences between included and excluded patients, the potential for selection bias arising from complete‐case exclusion cannot be excluded. Furthermore, the 12 months follow‐up—while adequate for capturing functional outcomes using mRS and CASE—may be insufficient to characterize very‐late relapses or subtle persistent cognitive deficits, particularly given that MoCA was not systematically repeated as a longitudinal outcome measure.

## 6 Conclusions

This study demonstrates that anti‐LGI1 encephalitis frequently presents with isolated cognitive impairment, with the majority of patients subsequently evolving into a broader neuropsychiatric syndrome. Crucially, a distinct subset of patients maintains sustained isolated cognitive impairment throughout the disease course, in which patients tended to be younger, exhibited relatively preserved naming, and frequently showed EEG and mesial temporal MRI abnormalities. Recognizing this clinical trajectory and integrating bedside cognitive features with supportive paraclinical findings may raise clinical suspicion and prompt timely antibody testing, thereby mitigating misdiagnosis as neurodegenerative dementia. However, because this cohort lacked an antibody‐negative comparison group, the diagnostic accuracy of these features could not be quantified; they should therefore be viewed as suspicion‐raising clues rather than validated diagnostic markers. Moreover, functional recovery assessed by global scales does not preclude residual cognitive deficits, underscoring the need for prospective studies incorporating comprehensive neuropsychological batteries and extended follow‐up to refine the cognitive fingerprint of this treatable mimic.

## Author Contributions

Y. Jia was the major contributors in writing the manuscript. Y. Jia, H. Wang, J. Zhang, and Y. Wang conceptualized the study. H. Wang, J. Zhang, and Q. Wang evaluated the patient's cognitive function and conducted follow‐up visits. H. Wang, J. Zhang, Y. Yang, L. Gao, W. Wang, J. Chen, Y. Lin collected samples and analyzed the clinical data. H. Kang, X. Su, and Z. Huang analyzed the brain MRI and EEG recordings. Z. Huang, L. Ren, A. Liu, and Y. Wang contributed to the diagnosis and treatment of patients. Wang YP accepts responsibility for conduct of research, final approval, and study supervision. All authors read and approved the final manuscript.

## Funding

This work was supported by National Natural Science Foundation of China [Grant No. 82401705], National Natural Science Foundation of China [Grant No. 82371461], National Key Research and Development Program of China [Grant No. 2022YFC2503802], and Department‐level Training Project of Capital Medical University Xuanwu Hospital [Grant No. QNPY202311].

## Consent

Written informed consent for publication was obtained from all participants or their legally authorized representatives. Written informed consent was obtained from all participants or their legally authorized representatives. The study protocol was approved by the Ethics Committee of Xuanwu Hospital, Capital Medical University (2019–015) and was conducted in accordance with the principles of the Declaration of Helsinki. The study is reported according to the Strengthening the Reporting of Observational Studies in Epidemiology (STROBE) reporting guideline.

## Conflicts of Interest

The authors declare no conflicts of interest.

## Supporting information


**Figure S1:** Flow diagram of patient screening and enrollment. A total of 125 patients with anti‐LGI1 encephalitis were screened across three participating centers. Eighty‐two patients were excluded due to failure to meet the inclusion criteria, and the remaining 43 patients were finally enrolled.
**Figure S2:** Positive LGI1 autoantibodies in serum and cerebrospinal fluid (CSF) from patients with cognitive impairment as the initial manifestation. Cell‐based immunofluorescence assays (Euroimmun) demonstrate LGI1 autoantibody positivity in serum (left panel) and cerebrospinal fluid (CSF, right panel) samples from a representative patient in the cohort study. In these assays, LGI1‐expressing cells were incubated with patient samples, followed by detection with an Alexa Fluor 488–conjugated secondary antibody (green). The cells show characteristic membrane immunofluorescence staining in both specimens. The illustrated case had a serum LGI1 antibody titer of 1:32 and a CSF titer of 1:100. All images were captured using standard immunofluorescence microscopy.
**Table S1:** The clinical information of LGI1 patients in the cohort study.
**Table S2:** Association between cognitive dysfunction assessed by MoCA and MRI/CSF biomarkers in patients with LGI1 encephalitis.

## Data Availability

Data are available upon reasonable request. The data that support the findings of this study are available from the corresponding author upon reasonable request.
