# Supplementary material for: The Clinical Profile and Bedside Clues of Anti‐LGI1 Encephalitis Presenting With Isolated Cognitive Impairment: A Multicenter Retrospective Cohort Study
Source: CNS Neurosci Ther. 2026 Nov 9;32(11):e71258. doi: 10.1002/cns.71258 (PMC13746320; doi:10.1002/cns.71258)
Supplement: Supplementary file 1 [file CNS-32-e71258-s001.docx]

**Figure S1. Flow diagram of patient screening and enrollment.** A total of 125 patients with anti-LGI1 encephalitis were screened across three participating centers. Eighty-two patients were excluded due to failure to meet the inclusion criteria, and the remaining 43 patients were finally enrolled.

**Figure S2. Positive LGI1 Autoantibodies in Serum and Cerebrospinal Fluid (CSF) From Patients With Cognitive Impairment as the Initial Manifestation.** Cell-based immunofluorescence assays (Euroimmun) demonstrate LGI1 autoantibody positivity in serum (left panel) and cerebrospinal fluid (CSF, right panel) samples from a representative patient in the cohort study. In these assays, LGI1-expressing cells were incubated with patient samples, followed by detection with an Alexa Fluor 488‒conjugated secondary antibody (green). The cells show characteristic membrane immunofluorescence staining in both specimens. The illustrated case had a serum LGI1 antibody titer of 1:32 and a CSF titer of 1:100. All images were captured using standard immunofluorescence microscopy.

**Table S1. The clinical information of LGI1 patients in the cohort study**

| **Characteristic** | | **LGI Encephalitis with Isolated Cognitive impairment-Onset Phenotype**  **(n=43)** |
| --- | --- | --- |
| Age at onset (years) [M (IQR)] | | 58 (17) |
| Gender (male), N (%) | | 29(67.4%) |
| MOCA, [M (IQR)] | | 17(9) |
| Degree of cognitive impairment (MOCA), N (%) | Mild cognitive impairment  (18-25 points) | 19(44.2%) |
|  | Moderate cognitive impairment  (10-17 points) | 18(41.9%) |
|  | Severe cognitive impairment  (Under 10 points) | 6(14.0%) |
| APE2 | APE2 scores | 5.0±2.1 |
|  | APE2 ≥ 4, N(%) | 31(72.1%) |
| CSF inflammatory abnormality^a^ | | 14(32.6%) |
| Antibody levels in CSF, N (%) | weakly positive | 11(25.6%) |
|  | moderately positive | 11(25.6%) |
|  | strongly positive | 21(48.8%) |
| Brain MRI abnormality, N (%) | Suggesting encephalitis^b^ | 21(48.8%) |
|  | Hippocampal atrophy | 6(14.0%) |
| EEG abnormality, N (%) | Slow wave | 16(37.2%) |
|  | Epileptic discharge | 17(39.5%) |
| Good recovery | | 36(83.7%) |
| Poor recovery | | 7(16.3%) |

a: CSF inflammatory abnormality was defined as is defined as the presence of at least one of the following abnormalities, including pleocytosis, elevated protein concentration, elevated immunoglobulin G index, or positive oligoclonal bands. b: Brain MRI hyperintense signals on T2WI / FLAIR sequences restricted to one or both medial temporal lobes or in multifocal areas involving grey matter, which highly suggested the features of autoimmune encephalitis.

**Table S2. Association Between Cognitive Dysfunction Assessed by MoCA and MRI/CSF Biomarkers in Patients with LGI1 Encephalitis**

| **Patients** | **Impaired Cognitive Domains** | | | | | | | | **Brain MRI and CSF analysis** | | |
| --- | --- | --- | --- | --- | --- | --- | --- | --- | --- | --- | --- |
|  | **Visuospatial abilities** | **Executive function** | **Naming** | **Attention** | **Language** | **Abstraction** | **Delayed Recall** | **Orientation** | **MRI mesiotemporal hyperintensities** | CSF inflammatory abnormality**^a^** | **CSF antibody titer levels^b^** |
| 1 |  |  |  |  |  |  | ● | ● | B | ● | S |
| 2 | ● | ● |  | ● | ● | ● | ● | ● |  |  | S |
| 3 |  |  |  |  |  |  | ● |  |  | ● | W |
| 4 | ● | ● | ● | ● | ● | ● | ● | ● | B | ● | S |
| 5 |  |  |  |  |  |  |  | ● | B |  | S |
| 6 |  |  |  |  |  |  | ● |  |  |  | W |
| 7 |  |  |  | ● |  |  | ● | ● |  |  | S |
| 8 |  |  |  | ● |  |  | ● |  | L |  | M |
| 9 |  |  |  | ● |  |  |  |  | B | ● | M |
| 10 | ● |  |  | ● |  |  | ● |  | R | ● | M |
| 11 |  |  |  | ● |  |  | ● | ● |  |  | W |
| 12 |  |  |  |  |  |  | ● |  |  | ● | M |
| 13 | ● |  |  | ● |  |  | ● |  | B |  | S |
| 14 |  |  |  | ● |  |  | ● |  |  |  | W |
| 15 |  |  |  |  |  |  | ● |  |  |  | M |
| 16 |  |  |  | ● | ● |  | ● |  | L |  | S |
| 17 | ● | ● | ● | ● | ● | ● | ● | ● |  |  | S |
| 18 |  | ● |  | ● |  |  | ● | ● |  | ● | W |
| 19 |  |  |  | ● |  |  | ● |  | B | ● | S |
| 20 | ● | ● | ● | ● | ● | ● | ● | ● |  | ● | M |
| 21 | ● | ● | ● |  |  | ● | ● | ● | B | ● | S |
| 22 |  |  |  |  |  |  | ● |  | L |  | S |
| 23 | ● |  |  |  |  |  | ● | ● | B |  | S |
| 24 | ● | ● | ● |  |  |  | ● | ● | B |  | S |
| 25 | ● | ● | ● |  |  |  | ● | ● |  |  | S |
| 26 |  |  |  |  |  |  | ● |  | B |  | M |
| 27 |  |  |  |  |  |  | ● | ● |  |  | S |
| 28 | ● | ● | ● | ● | ● |  | ● | ● | B |  | W |
| 29 | ● | ● |  |  |  |  | ● | ● | R |  | W |
| 30 |  |  |  |  |  |  |  | ● |  |  | W |
| 31 |  |  | ● | ● |  |  | ● | ● |  |  | W |
| 32 |  |  |  |  |  |  | ● | ● | L |  | S |
| 33 | ● | ● | ● | ● | ● | ● | ● | ● |  | ● | S |
| 34 | ● | ● | ● | ● |  |  | ● | ● |  |  | W |
| 35 | ● | ● | ● | ● |  | ● | ● | ● | L |  | S |
| 36 |  |  |  | ● |  | ● | ● |  | B |  | S |
| 37 | ● | ● |  |  | ● |  | ● |  | B | ● | S |
| 38 | ● | ● |  | ● | ● |  | ● | ● |  | ● | M |
| 39 | ● | ● |  |  | ● |  | ● | ● |  |  | S |
| 40 |  |  |  | ● |  |  | ● | ● | B |  | M |
| 41 |  |  |  | ● |  |  | ● |  |  | ● | M |
| 42 |  |  |  | ● |  |  | ● |  |  |  | W |
| 43 |  |  |  | ● |  |  | ● | ● |  |  | M |

a: CSF inflammatory abnormality was defined as is defined as the presence of at least one of the following abnormalities, including pleocytosis, elevated protein concentration, elevated immunoglobulin G index, or positive oligoclonal bands.

b: The initial dilution titers for CSF was 1:1. CSF antibody titers were categorized as weakly positive (1:1), moderately positive (1:3.2 to 1:10), or strongly positive (≥1:32).

MRI: magnetic resonance imaging; B: bilateral; L: left; R: right; W: weakly positive; M: moderately positive; S: strongly positive
